# The virtual reference radiologist: comprehensive AI assistance for clinical image reading and interpretation

**DOI:** 10.1007/s00330-024-10727-2

**Published:** 2024-04-16

**Authors:** Robert Siepmann, Marc Huppertz, Annika Rastkhiz, Matthias Reen, Eric Corban, Christian Schmidt, Stephan Wilke, Philipp Schad, Can Yüksel, Christiane Kuhl, Daniel Truhn, Sven Nebelung

**Affiliations:** Department of Diagnostic and Interventional Radiology, University Hospital RWTH Aachen, Aachen, Germany

**Keywords:** Radiology, Diagnostic imaging, Artificial intelligence, Diagnostic errors

## Abstract

**Objectives:**

Large language models (LLMs) have shown potential in radiology, but their ability to aid radiologists in interpreting imaging studies remains unexplored. We investigated the effects of a state-of-the-art LLM (GPT-4) on the radiologists’ diagnostic workflow.

**Materials and methods:**

In this retrospective study, six radiologists of different experience levels read 40 selected radiographic [*n* = 10], CT [*n* = 10], MRI [*n* = 10], and angiographic [*n* = 10] studies unassisted (session one) and assisted by GPT-4 (session two). Each imaging study was presented with demographic data, the chief complaint, and associated symptoms, and diagnoses were registered using an online survey tool. The impact of Artificial Intelligence (AI) on diagnostic accuracy, confidence, user experience, input prompts, and generated responses was assessed. False information was registered. Linear mixed-effect models were used to quantify the factors (fixed: experience, modality, AI assistance; random: radiologist) influencing diagnostic accuracy and confidence.

**Results:**

When assessing if the correct diagnosis was among the top-3 differential diagnoses, diagnostic accuracy improved slightly from 181/240 (75.4%, unassisted) to 188/240 (78.3%, AI-assisted). Similar improvements were found when only the top differential diagnosis was considered. AI assistance was used in 77.5% of the readings. Three hundred nine prompts were generated, primarily involving differential diagnoses (59.1%) and imaging features of specific conditions (27.5%). Diagnostic confidence was significantly higher when readings were AI-assisted (*p* > 0.001). Twenty-three responses (7.4%) were classified as hallucinations, while two (0.6%) were misinterpretations.

**Conclusion:**

Integrating GPT-4 in the diagnostic process improved diagnostic accuracy slightly and diagnostic confidence significantly. Potentially harmful hallucinations and misinterpretations call for caution and highlight the need for further safeguarding measures.

**Clinical relevance statement:**

Using GPT-4 as a virtual assistant when reading images made six radiologists of different experience levels feel more confident and provide more accurate diagnoses; yet, GPT-4 gave factually incorrect and potentially harmful information in 7.4% of its responses.

## Key Points

- *The benefits and dangers of GPT-4 for textual assistance in radiologic image interpretation are unclear.*
- *GPT-4’s textual assistance improved radiologists’ diagnostic accuracy from 75 to 78%.*
- *Less experienced radiologists used GPT-4 for guidance on differential diagnoses and imaging findings.*

## Introduction

Large language models (LLMs) are steadily advancing in various sectors, including healthcare. As the first mainstream dialogue-based artificial intelligence (AI) model, chatGPT has gained immense popularity [1]. Even though prior LLMs, such as BERT (Bidirectional Encoder Representations from Transformers), gained popularity in the past [2], attention transformer-based LLMs, such as chatGPT, have largely replaced them.

Potential use cases involve radiologic reporting [3, 4] and guidance on utilizing imaging services [5]. ChatGPT passed the United States Medical Licensing Exam [6] and nearly passed a radiology board-style examination without images [7]. Recent studies have summarized LLMs’ evolving role and impact in radiology. Bajaj et al highlighted the potential of LLMs to improve image interpretation efficiency and streamline radiologists’ workflows [8]. D’Antonoli et al emphasized the need for radiologists to understand their technical basics, ethical considerations, and potential risks [9]. Bera et al analyzed the available literature on ChatGPT (as of August 2023) and found 51 studies that detailed the model’s multifaceted applications in radiology and the -by and large- “impressive performance” [10]. Specifically, ChatGPT’s capability to evaluate patient studies and provide radiologic diagnoses has been studied in well-presented literature case series such as the American Journal of Neuroradiology’s “Case of the Month” [11] and Radiology’s “Diagnosis Please” series [12]. ChatGPT was also fed appropriateness criteria (by the American College of Radiology) to create a context-aware chatbot for improved decision-making for clinical imaging [13]. Yet, the tool’s potential to provide “reading room assistance” to the radiologist when reading and interpreting imaging studies has not been evaluated. Our objectives were (i) to investigate chatGPT’s effects on diagnostic accuracy and confidence, (ii) to study user interactions with chatGPT, and (iii) to assess the quality of chatGPT’s responses in terms of accuracy, up-to-dateness, and reliability. Our hypothesis was that radiologists would benefit from chatGPT’s assistance, particularly when inexperienced. Here, we implicitly operated under the null hypothesis (H_0_) of no difference in diagnostic accuracy and confidence with and without AI assistance. Conversely, the alternative hypothesis (H_1_) assumed that AI assistance provides a measurable difference.

## Materials & methods

### Study design and dataset characteristics

Approval was granted by the local ethical committee (reference number 028/19), and the requirement to obtain individual informed consent was waived.

**Fig. 1:**
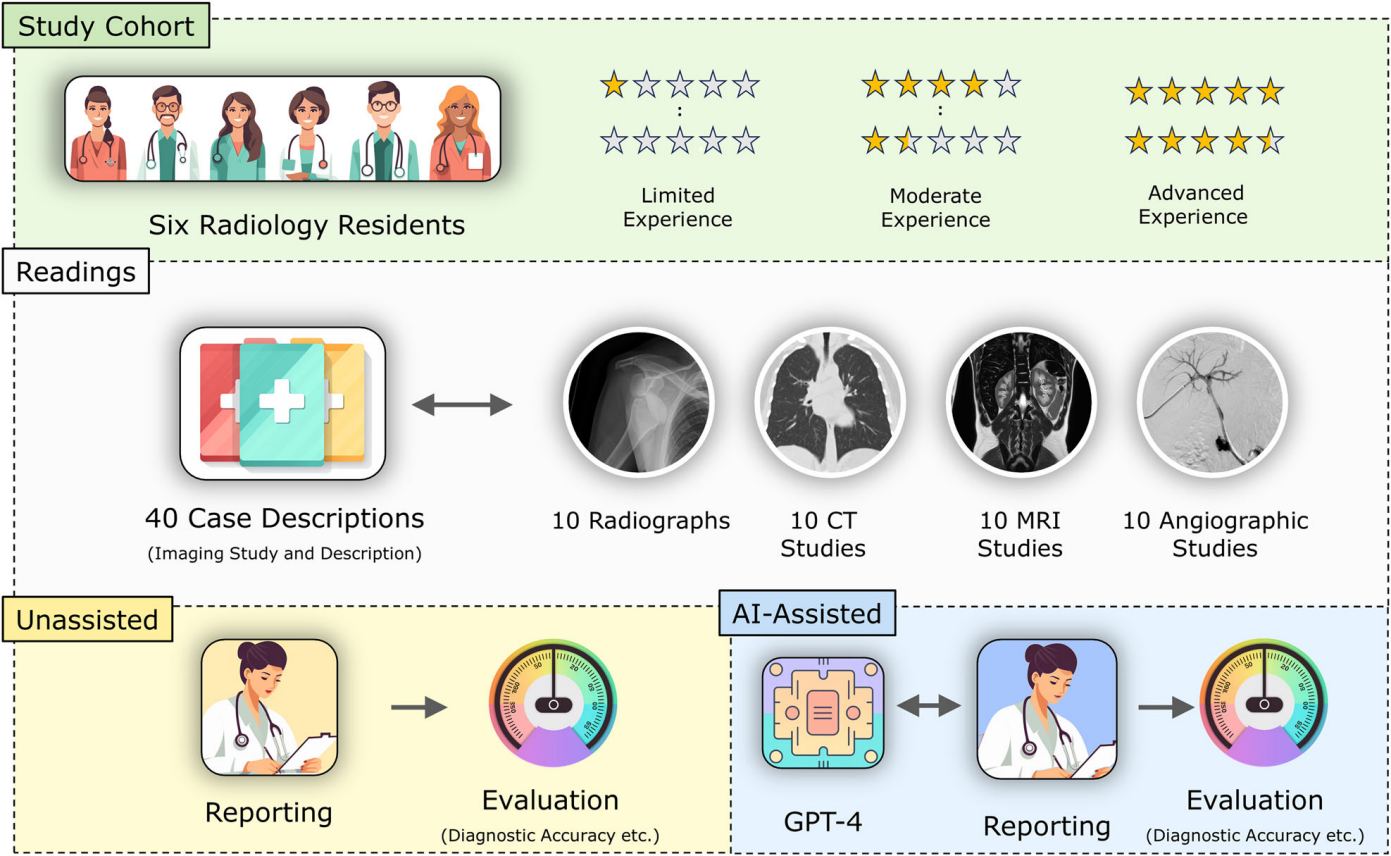
Study workflow. Six radiology residents of variable experience levels read 40 imaging studies comprising clinical information and images under unassisted and AI-assisted clinical conditions. Their differential diagnoses were evaluated regarding diagnostic accuracy and confidence. Stars indicate years of experience

This study was designed as a retrospective intra-individual comparative reader study on existing imaging studies that were prospectively read with and without assistance by GPT-4, the latest version of ChatGPT, to evaluate its effects on the radiologic workflow. Figure 1 details the study workflow.

### Selection of imaging studies

By screening the local PACS (Picture Archiving and Communication System, iSite, Philips Healthcare, Best, Netherlands) of our tertiary academic medical center (University Hospital Aachen, Aachen, Germany), two resident radiologists with three years of experience (R.S. and M.H.) and two board-certified clinical radiologists (D.T. and S.N. [with 10 and 8 years of experience]) selected ten radiographic, CT, MRI, and angiographic studies each (Table 1). The imaging studies reflected various demographic characteristics, i.e., patient age and sex, and conditions of variable severity and complexity. Only studies with unequivocal findings were selected, and the reference diagnoses were established based on the synopsis of the original radiologic reports, associated clinical and nonimaging findings, and follow-up studies. Imaging studies were disregarded in the case of inconsistent or contradictory findings. After anonymization, patients’ age, sex, and other details, such as the reason for the exam, were removed from the studies, and only the study to be assessed was included. Angiographic studies, for example, were prepared so that the postdiagnostic therapeutic and post-therapeutic image series were removed. The studies were fed back to the research section of the PACS individually, where they could be accessed on clinical workstations. Standardized case descriptions were framed for each study, indicating the relevant clinical context and allowing radiologists to put their findings into the appropriate clinical perspective.

**Table 1:** Details of the imaging studies

Modality	Diagnosis (reference)	Case description
**Radiography**	Osgood-Schlatter Disease (Apophysitis of Tibial Tubercle)	13 yo male with pain and swelling over the tibial tuberosity exacerbated with exercise.
	Osteopetrosis	49 yo male with recurrent bone fractures and infections.
	Rickets	9 yo female with recurrent bone fractures.
	Duodenal Atresia	1-day-old male with nonbilious vomiting.
	Kienböck’s Disease (Lunate Malacia)	31 yo male patient with wrist pain.
	Cystic Fibrosis	17 yo male with recurrent infections, wheezing, coughing, and shortness of breath.
	Interstitial Pneumonia	61 yo male with chest pain and dyspnea three days after amputation of the right lower leg.
	Pulmonary Alveolar Microlithiasis	55 yo male with persistent cough and dyspnea, especially during physical exertion.
	Segond Fracture	48 yo male with knee pain and swelling after trauma.
	Miliary Tuberculosis	25 yo male homeless person with weight loss, fever, weakness, dyspnea, and hypercalcemia.
**CT**	Sarcoidosis	36 yo male with persistent dry cough, shortness of breath, fatigue, and ankle pain.
	Interstitial Pancreatitis (Uncomplicated)	81 yo female with upper abdominal pain radiating to the back.
	Endocrine Orbitopathy	51 yo male with eye pain. His family says his eyes look ‘weird’.
	Intralobar Pulmonary Sequestration	32 yo female with recurrent pulmonary infections since childhood.
	Gall Stone Ileus	66 yo male with nausea, vomiting, abdominal distention, and cramping abdominal pain.
	Sigmoid Volvulus	18 yo male with abdominal pain, abdominal distension, vomiting, and bloody stools.
	Portal Vein Thrombosis	24 yo female with sudden acute abdomen, fever, diarrhea, and hepatosplenomegaly.
	Bronchogenic Cyst	84 yo female with cough, stridor, shortness of breath, and recurrent upper respiratory infections.
	Ruptured Echinococcus Cyst	71 yo female with a fever, upper abdominal pain, and calcified liver mass on ultrasound.
	Left Coronary Artery Anomaly	18 yo male with intermittent chest pain and dyspnea.
**MRI**	Lipoma Arborescens (Knee)	41 yo female with recurrent joint effusions of both knees.
	Nutcracker Syndrome with Pelvic Congestion	31 yo female with recurrent pelvic pain after prolonged sitting and sexual intercourse.
	Iron Overload (Sickle Cell Anemia)	18 yo male with anemia and recurrent acute bone pain.
	Hoffa Pad Impingement Syndrome	24 yo female with unspecific knee pain.
	Focal Nodular Hyperplasia (Liver)	38 yo female with an incidental liver mass on ultrasound.
	Primary Sclerosing Cholangitis (Liver)	65 yo male with recurrent bloody diarrhea and abdominal pain.
	Hemangioma (Spleen)	66 yo patient with unclear spleen finding in ultrasound. Established diagnosis of adenoma of the liver and adenomyomatosis of the gallbladder.
	Giant Cell Arteritis (Head)	80 yo male with headache, fever, blurred vision, and jaw pain during mastication.
	Ruptured Baker Cyst (Knee)	62 yo male with sudden knee pain and swelling.
	Breast Cancer Left (Triple-negative, No Special Type)	34 yo female with a new firm mass in the breast.
**Angiography**	Tumor Blush (Hepatocellular Carcinoma)	74 yo male with cirrhosis and liver mass of unknown origin.
	Active Bleeding (Accessory Renal Artery)	72 yo male with hypotension, tachycardia, paleness, and right flank pain.
	Bile Leakage (Insufficiency of the Percutaneous Transhepatic Biliary Drainage)	69 yo male after hepatobiliary surgery and percutaneous transhepatic biliary drainage. Increasing markers of inflammation and fever.
	Peripheral Artery Disease (Proximal Stenosis of Superficial Femoral Artery)	54 yo male with leg pain during physical activity that resolves with rest.
	Thrombosis (Port Catheter Tip)	75 yo female with port catheter dysfunction.
	Active Bleeding (Shunt Vein Rupture)	86 yo male with sudden excruciating pain in the left axilla. Dialysis shunt in the left arm.
	Thrombosis (Femoral Vein with Postthrombotic Changes)	30 yo male with pain and swelling of the left leg.
	Transjugular Intrahepatic Portosystemic Shunt Occlusion	48 yo male with ascites one year after Transjugular Intrahepatic Portosystemic shunting due to liver cirrhosis.
	Primary Sclerosing Cholangitis	41 yo female with recurrent cholestasis, itching, and upper abdominal pain 4.5 years after liver transplantation.
	Subtotal Middle Cerebral Artery Occlusion	69 yo male with sudden left-sided hemiparesis and aphasia.

For each modality, 10 representative imaging studies were collected and complemented with patient descriptions to allow for establishing an unequivocal diagnosis.

*yo* year(s)-old

### Experimental setup and data collection

Six clinical resident radiologists with varying experience levels were recruited. Because the radiology residency in Germany usually takes five years, their experience level was trichotomized as limited (up to 1 year of clinical experience), moderate (between 1 and 4 years of clinical experience), and advanced (more than 4 years of clinical experience but not yet board certified). Two radiologists of each experience level were recruited.

The radiologists were asked to diagnose each patient based on the imaging study and case description in two sessions: (i) unassisted and (ii) AI-assisted. “Unassisted” meant that external references, e.g., online searches or textbooks, were prohibited. “AI-assisted” meant that GPT-4 could be prompted without restrictions. Additional external references were similarly prohibited from singling out the effect of GPT-4. We did not use any additional GPT-4 add-ons, meaning GPT-4 had to resort to its internal knowledge. The radiologists were introduced to the setup and adequately trained to interact with GPT-4. They were also instructed that prompts could query any aspect of diagnostic decision-making, from clinical diagnoses and associated imaging findings to radiologic signs of diseases and gradings to classifications. To assess the natural interaction with the tool, no guidance on optimizing the interaction or identifying “hallucinations” was provided.

The imaging studies were read on in-house radiology workstations. Per case, radiologists provided up to three diagnoses, ranked in descending order of probability, and the confidence level using a five-point Likert scale ranging from “very unsure” (score 1) to “very sure” (score 5). After completing the questionnaires unassisted (session one), the radiologists re-read the same studies using AI assistance (session two). Time restrictions or a minimum washout period were not instituted, and the radiologists could re-read the imaging studies at their chosen time. However, they were instructed not to collect additional information or seek assistance on the patients, studies, or differential diagnoses between the readings. Further details on the experimental setup are provided in the Supplementary Text and Supplementary Fig. 1.

### Outcome metrics and evaluation

Different aspects of the diagnostic workflow were evaluated as performance, interaction, and user experience metrics.

### Performance

Diagnostic accuracy was assessed based on the radiologist-provided diagnoses and counted as correct if the correct diagnosis was (i) among the three differential diagnoses (‘top-3 performance’) and (ii) the first differential diagnosis (‘top-1 performance’). Superordinate diagnoses (e.g., ‘peripheral artery disease’) instead of the more specific diagnosis (e.g., ‘superficial femoral artery stenosis’) were considered correct if the clinical presentation and imaging findings did not differ considerably. Overly vague diagnoses (like ‘vasculopathy’) or considerably different clinical presentations and imaging findings (like ‘tuberculosis’ instead of ‘miliary tuberculosis’) were rejected. Ambiguous diagnoses were discussed by the four radiologists above and considered against the case description and imaging findings.

### Interaction

The radiologist-GPT-4 interactions were parameterized and quantified as the number and type of prompts per study. Prompts were categorized based on their purpose as (i) asking for differential diagnoses, (ii) seeking clarification on gradings and classifications, (iii) requesting pathology-related information, (iv) requesting anatomy-related information, (v) enquiring about imaging features of a condition, or (vi) asking for basic guidance on how to interpret a particular imaging study. Prompts could be assigned to different prompt types. Response quality was assessed qualitatively, and four radiologists (M.H., R.S., D.T., S.N.) evaluated whether the information provided by GPT-4 was verified, up-to-date, and reliable. They analyzed all prompts and responses independently and noted possibly incorrect or inconsistent responses that were subsequently discussed until a consensus was reached. ‘hallucinations’ were defined as seemingly correct responses that (i) were nonsensical when considered against common knowledge in radiology or (ii) inconsistent with framework information or conditions stated in the radiologist’s request. ‘Misinterpretations’ were defined as GPT-4 misunderstanding a question or providing contextually misleading or irrelevant responses. ‘Clarifications’ were defined as GPT-4 lacking understanding of a prompt that necessitated its rephrasing. Notably, the training of GPT-4 was concluded in 2021, which was considered when evaluating the response quality.

### User experience

Diagnostic confidence (with and without AI assistance) was registered as above. Additionally, the radiologists were asked to provide general feedback on satisfaction, utility, ease of use, and trust on five-point Likert scales, ranging from ‘very poor’ (score 1) to ‘very good’ (score 5).

### Statistical analysis and power analysis

Statistical analyses were performed using GraphPad Prism software (v9.5, San Diego, CA, USA) and Python (v3.11) and its library *statsmodel* by R.S., M.H., D.T., and S.N. Diagnostic accuracy was calculated as the number of correct diagnoses divided by the number of correct and incorrect diagnoses for the ‘top-3 performance’ and ‘top-1 performance’ approaches. We used a generalized linear mixed-effects model within a logistic regression framework to account for the binary outcome, i.e., correct and incorrect. Experience, modality, and AI assistance were treated as fixed effects and the radiologists as random effects. After yielding inflated coefficients, likely due to overfitting and multicollinearity, we used a simplified model that focused exclusively on the main predictors of diagnostic accuracy. An analogous model was used to study the predictors’ impact on diagnostic confidence. For the top-3 and the top-1 performance approaches, a two-proportion z-test was used to determine whether differences in diagnostic accuracy were significant between AI-assisted and unassisted radiologists. Post hoc, the effect size was quantified using Cohen’s *h* as a measure of the difference between two proportions. Means and 95% confidence intervals are given, and the significance level was set at α ≤ 0.05.

Given the scarce availability of literature evidence on diagnostic accuracy as a function of AI assistance, a rudimentary sample size estimation was conducted. Informed by related literature evidence [14], we assumed a small effect size of 0.2. Consequently, the minimum sample size was determined a priori as 208 using the power of 0.8, the probability of an α error of 0.05, a t-test and Wilcoxon signed-rank test (matched pairs), and a two-tailed procedure (G*Power, v3.1.9.7; Heinrich-Heine-University; [15]). Supplementary Figure 2 provides a screenshot of the sample size estimation.

## Results

The study was conducted between May 10th and June 5th, 2023. Our study involved a sample size of 240 studies, which exceeded the calculated minimum sample size required for adequate power, as determined by our prior power analysis. All radiologists completed the unassisted (session one) and AI-assisted (session two) readings during this period. The time delay between the reading sessions was 6.3 ± 6.3 days (range, 0–18 days).

**Fig. 2:**
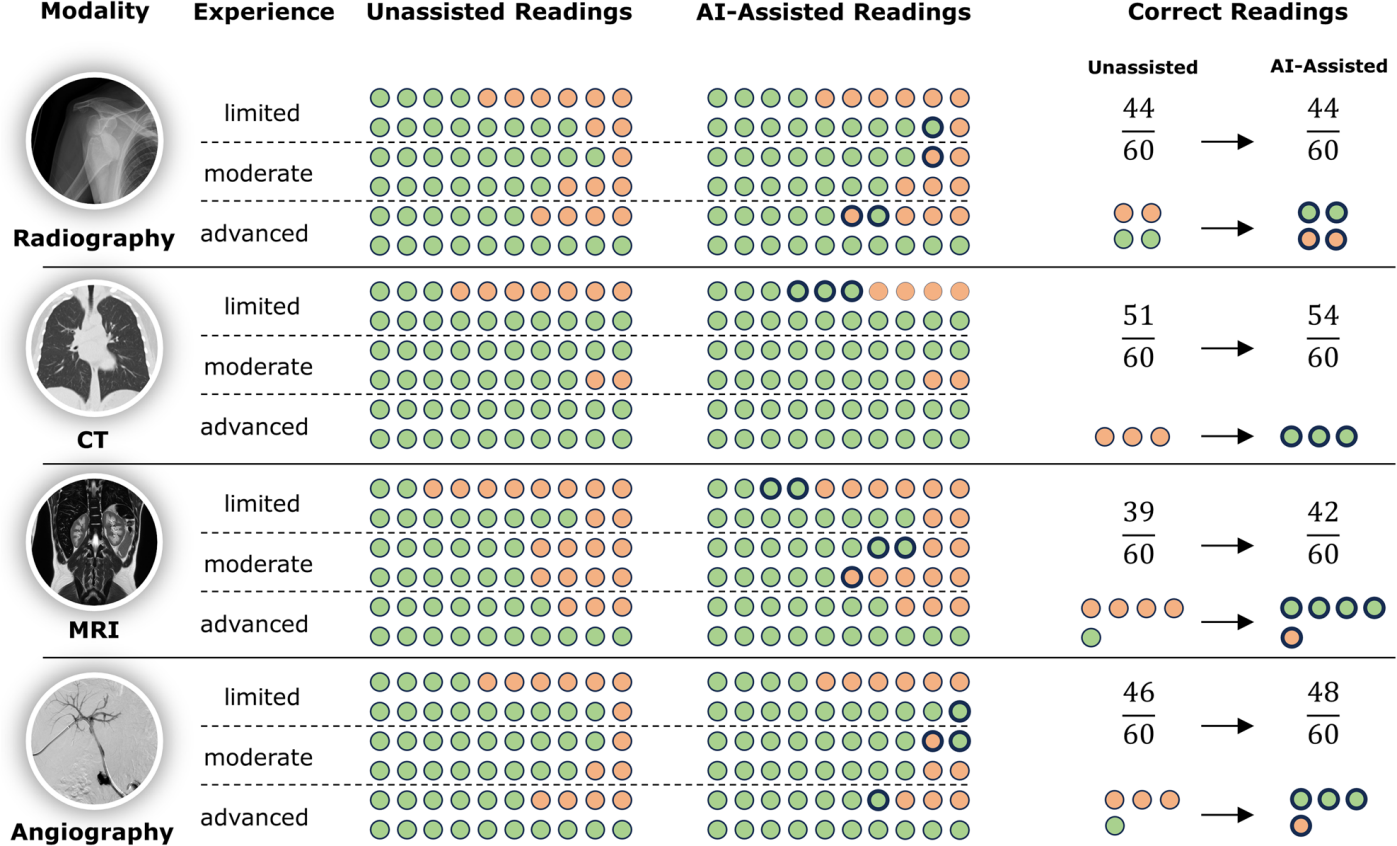
Diagnostic accuracy as a function of modality, experience level, and AI assistance. Detailed breakdown of the correct and incorrect readings per imaging study when considering the correct diagnosis among the top three radiologist-provided diagnoses (‘top-3 performance’). Green circles indicate correct diagnoses, and red circles incorrect diagnoses. Bold circles indicate diagnoses that changed using AI

**‘**Top-3 performance’: we found moderately improved diagnostic accuracy when considering the three radiologist-provided diagnoses. Specifically, accuracy improved from 181/240 (75.4%, unassisted) to 188/240 studies (78.3%, AI-assisted), which aligns with the expected effect direction and magnitude. Yet, the hypothesis that radiologists’ diagnostic accuracy would benefit from using AI assistance cannot be accepted based on the current study, as the calculated effect size for diagnostic accuracy improvement was small (Cohen’s h: 0.069 [top-3] and 0.079 [top-1]) and not statistically significant (*p* = 0.130 [top-3] and *p* = 0.083 [top-1]; z-test). The greatest increases in correct diagnoses were found for radiologists with low experience and CT and MRI (Fig. 2), which supports our hypothesis (i). Supplementary Table 1 details the total counts of correct and incorrect diagnoses and diagnostic accuracy.

**Fig. 3:**
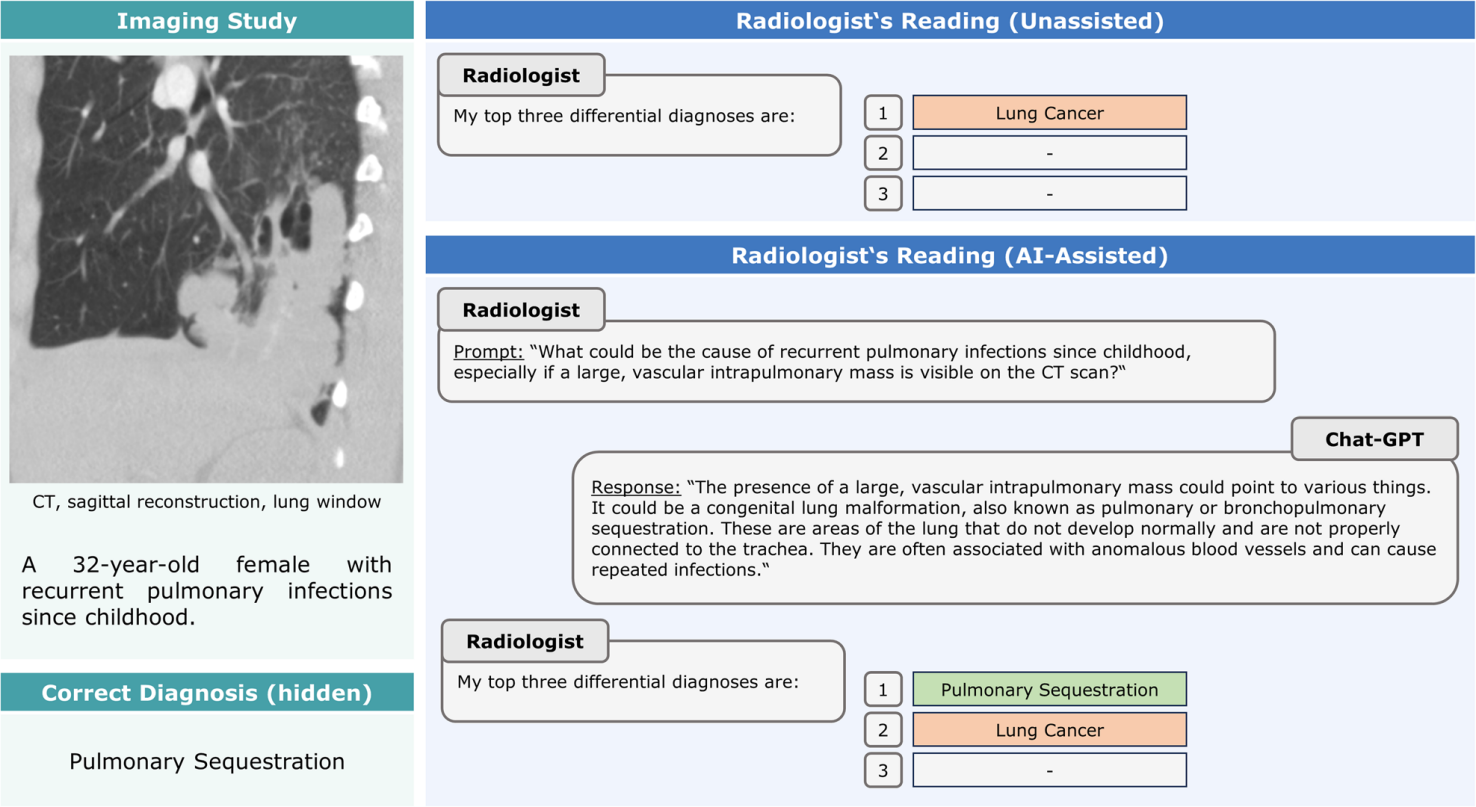
Positive effects of AI consultation—example case. In this patient with intralobar pulmonary sequestration (CT, sagittal reconstruction, lung window), the consultation of GPT-4 changed the initially incorrect differential diagnosis to the correct differential diagnosis

**Fig. 4:**
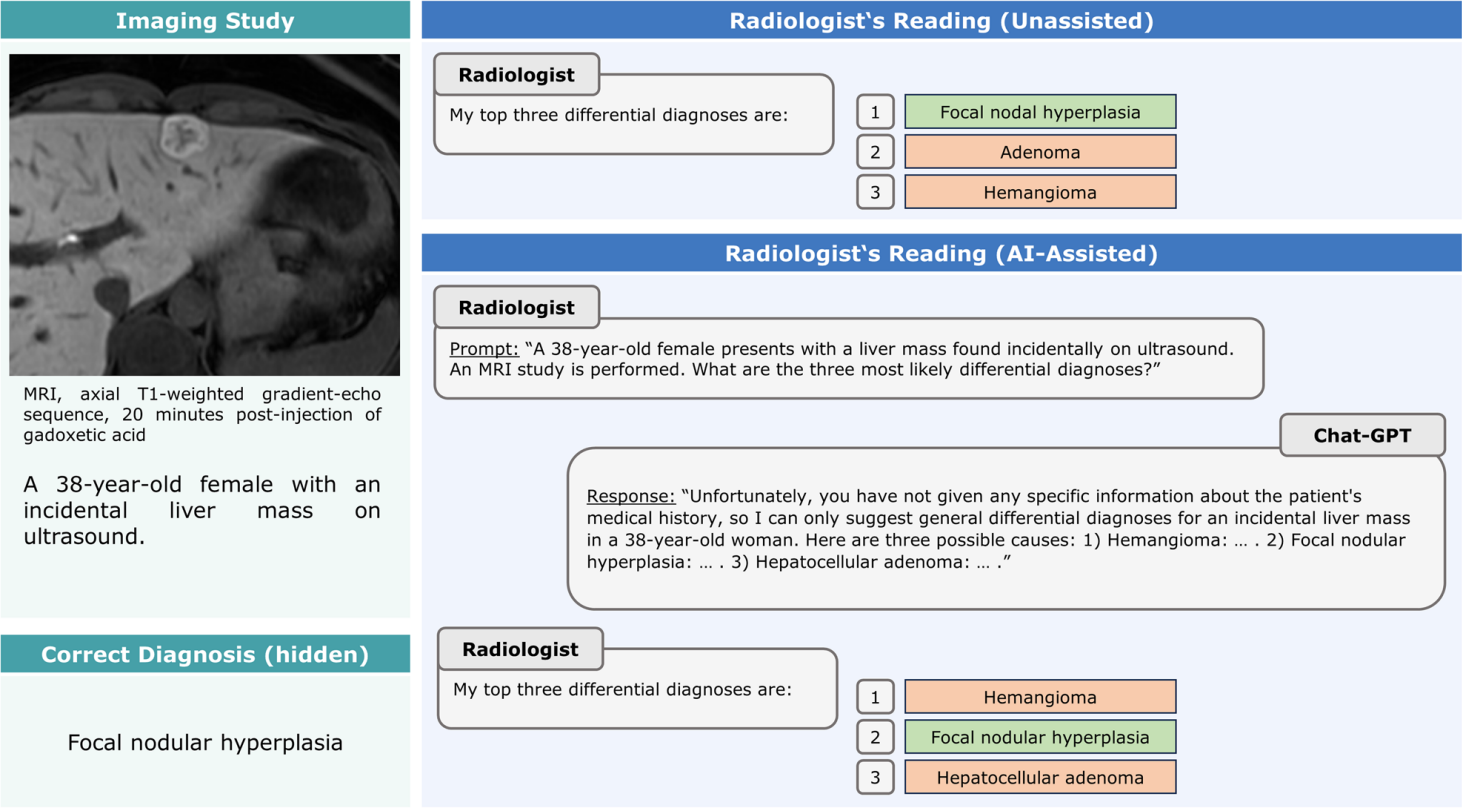
Negative effects of AI consultation—example case. In this patient with focal nodular hyperplasia (MRI, T1-weighted fat-suppressed gradient echo-sequence [Dixon]), axial image, 20 min after injection of gadoxetic acid), the consultation of GPT-4 changed the list and order of differential diagnoses. While focal nodular hyperplasia was the first differential diagnosis without AI assistance, it was only the second diagnosis with AI assistance, most likely because of adherence to the response

Most initial differential diagnoses remained unchanged despite AI assistance. However, in 12/240 re-read studies, initially incorrect differential diagnoses were revised after interaction with GPT-4 and rendered correct (Fig. 3). Conversely, in 4/240 re-read studies (two radiographic, one MRI, and one angiographic study), initially correct differential diagnoses (unassisted) were rendered incorrect (AI-assisted) (Fig. 4).

Statistically significant predictors of diagnostic accuracy were the experience levels. Radiologists with low and moderate experience were significantly less likely to provide correct diagnoses than radiologists with advanced experience (*p* ≤ 0.017). For modality and AI assistance, the effects were less clear. For AI assistance, the coefficient was -0.18 (*p* = 0.428), indicating a slightly yet nonsignificantly decreased likelihood of a correct diagnosis without AI assistance. Supplementary Table 2 details the coefficients and *p* values of the principal predictors that influenced diagnostic accuracy.

‘Top-1 performance’: when considering only the first radiologist-provided diagnoses, diagnostic accuracy improved slightly from 154/240 (64.2%, unassisted) to 163/240 studies (67.9%, AI-assisted) read correctly. Once again, the most pronounced improvements were found among radiologists with less experience (Fig. 2), which aligns with our hypothesized benefit of AI assistance for those radiologists. Supplementary Tables 2 and 3 detail the counts of correct and incorrect diagnoses and associated predictors for the ‘top-1’ performance.

### Prompt characteristics

**Fig. 5:**
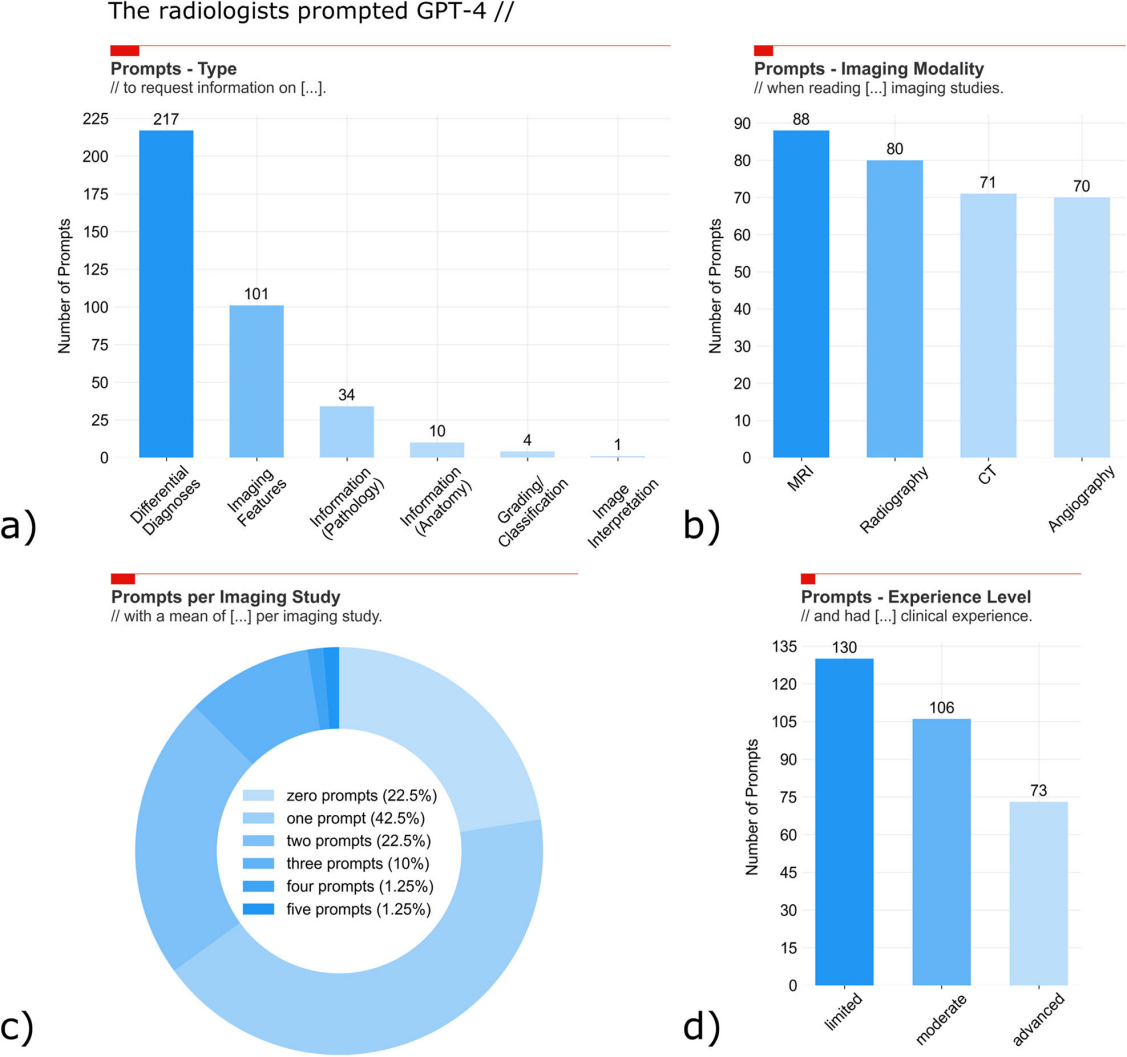
Prompt quantities and characteristics. During the AI-assisted readings of the imaging studies, the six radiologists provided *n* = 309 prompts altogether. Detailed are the prompt numbers (**a**, **b**, **d**) and percentages (**c**) regarding the prompt type (**a**), the imaging modality (**b**), the number of prompts per imaging study (**c**), and the radiologist’s experience level (**d**). Because prompts could be assigned to different type categories, the prompt sums differed between **a** versus **c** and **d**

Radiologists used GPT-4 in 77.5% of the studies, generating 309 prompts. Most prompts involved differential diagnoses (59.1% [*n* = 217]) and imaging features of specific conditions (27.5% [*n* = 101]). Less frequently, pathology- and anatomy-related information were requested (9.3% [*n* = 34] and 2.7% [*n* = 10]). Four prompts (1.3%) were related to gradings and classifications, and one prompt (0.3%) demanded general guidance (Fig. 5a). Prompts were evenly distributed among the modalities (Fig. 5b). Mainly, one prompt was used per patient (42.5% of prompts), while two (22.5%) or three prompts (10.0%) were used frequently, too (Fig. 5c). Fourty-two percentage of the prompts were provided by radiologists with limited experience, 34.3% by radiologists with moderate experience, and 23.6% by radiologists with advanced experience (Fig. 5d). Overall, the ample and variable interaction patterns of radiologists with GPT-4 reflect the expected natural engagement and confirm hypothesis (ii). Diagnostic confidence: radiologists were significantly more confident in their diagnoses when performing readings AI-assisted, regardless of the modality (*p* < 0.001) (Table 2). However, when considering experience levels, diagnostic confidence was significantly greater in radiologists of moderate experience only (versus advanced experience) (Supplementary Table 4).

**Table 2:** Diagnostic confidence scores as a function of AI assistance, imaging modality, and experience level

		Unassisted	AI-assisted
**All radiologists**	**Radiography**	3.17 ± 1.08	3.73 ± 0.97
	**CT**	3.53 ± 1.07	3.82 ± 1.10
	**MRI**	2.33 ± 1.10	2.93 ± 1.12
	**Angiography**	3.25 ± 1.08	3.77 ± 0.95
**Low experience**	**Radiography**	3.40 ± 1.19	4.00 ± 1.03
	**CT**	2.95 ± 1.15	3.70 ± 1.38
	**MRI**	2.35 ± 1.27	3.20 ± 1.28
	**Angiography**	3.45 ± 1.28	4.30 ± 0.86
**Moderate experience**	**Radiography**	3.50 ± 1.05	4.25 ± 0.72
	**CT**	4.30 ± 0.73	4.55 ± 0.60
	**MRI**	2.00 ± 0.97	3.10 ± 1.25
	**Angiography**	3.25 ± 0.85	4.05 ± 0.69
**Advanced experience**	**Radiography**	2.60 ± 0.75	2.95 ± 0.60
	**CT**	3.35 ± 0.81	3.20 ± 0.70
	**MRI**	2.65 ± 0.99	2.50 ± 0.61
	**Angiography**	3.05 ± 1.10	2.95 ± 0.69

Following each imaging study, radiologists indicated their diagnostic confidence on a semiquantitative Likert scale of very low (score 1) to very high (score 5). Data are mean ± standard deviation. The effects of experience level, imaging modality, and AI assistance were compared using a linear mixed-effect model. Its results are detailed in Supplementary Table 4

### Hallucinations

Twenty-three responses of GPT-4 were classified as ‘hallucinations’ (7.4%), and two as ‘misinterpretations’ (0.6%), while no ‘clarifications’ were necessary. Hallucinations involved all modalities and various aspects of imaging findings and their interpretation. Often, the hallucinations involved incorrect information on conditions and their imaging and nonimaging findings, e.g., opacity versus lucency of bone lesions (radiography), features of aortic dissection (CT), signal intensities and enhancement features of liver lesions (MRI), and vascular anatomy (angiography). GPT-4 occasionally disregarded the information provided in the prompt. Hallucinations did not detrimentally affect the list of differential diagnoses provided. Table 3 details and comments on the hallucinations found. Supplementary Table 5 details the ‘misinterpretations’. Even though the responses were largely consistent with current medical knowledge, the noted instances of hallucinations partially refute hypothesis (iii).

**Table 3:** ‘Hallucinations’ by GPT-4

Radiologist’s prompt	GPT -4’s hallucinatory response	Analysis of hallucination
[Radiography] 25-year-old male patient with weight loss, fever, weakness, dyspnea, and hypercalcemia. Finely speckled densities are observed bilaterally in the lungs. What are the most likely diagnoses?	[…] Tuberculosis: The classic symptoms of Tuberculosis are coughing, weight loss, night sweats, and fever. Lung effects can be visible on an X-ray. However, hypercalcemia is rather rare in Tuberculosis.	Hypercalcemia is common in patients with Tuberculosis [17].
[Radiography] What knee joint injury is consistent with a roundish opacity in the lateral tibial plateau?	[…] Cysts: Bone cysts, such as subchondral cysts, can also create opacities.	(Bone) cysts do not cause ‘opacities’ but ‘lucencies’.
[Radiography] What could explain the finding (i.e., Segond fracture) in the tibial plateau?	[…] Bone erosion: In certain conditions like rheumatoid arthritis, there can be bone erosions, which can present as opacities on the x-ray image.	Erosions do not cause ‘opacities’ but ‘lucencies’.
[Radiography] 31-year-old male patient with wrist pain. What are the most likely diagnoses (in sorted order) for densification of the os lunatum?	[…] Scaphoid cysts: These are relatively rare, but small fluid-filled sacs could cause densification in the x-ray image.	Bone cysts are lucent compared to bone and, thus, not a possible differential diagnosis in a patient with an opacified lunate.
[Radiography] In which disease does the double bubble sign occur? What are the probable differential diagnoses for non-bilious vomiting in newborns?	Non-bilious vomiting in newborns can have various causes, and some possible differential diagnoses include: Malrotation and Volvulus … Food Allergies or Intolerances … Infections …	Differential diagnoses do not consider the non-bilious quality of the vomiting (as stated in the prompt). Non-bilious vomiting implies an obstruction proximal to the ampulla of Vater.
[Radiography] What are the most probable differential diagnoses for a 49-year-old male patient with recurring bone fractures and infections? Compacted endplates of the vertebral bodies can be seen in the lumbar spine.	[…] Multiple Myeloma: This is a form of bone marrow cancer that can lead to bone pain, fractures, and infections. An X-ray image can show “densified” areas that are actually due to bone damage.	Multiple myeloma typically presents with hyperlucent (instead of “densified”) bone areas.
[Radiography] A uniform expansion and increased sclerosis of the vertebral body endplates can be seen across the entire spine, with the intervening vertebral body appearing comparatively less sclerotic. What is the associated radiologic sign called? What diseases?	The description suggests the so-called “sandwich vertebra” sign. […] This radiologic sign is typical for a disease called Chronic Recurrent Multifocal Osteomyelitis (CRMO), also known as Synovitis, Acne, Pustulosis, Hyperostosis, Osteitis (SAPHO) Syndrome. […]	The “sandwich vertebra” sign is not associated with CRMO but, in fact, with osteopetrosis.
[Radiography] Which diseases match fine speckled-confluent infiltrates on both sides with sparing of the periphery?	Potential differential diagnoses are […] Pneumonia […] Tuberculosis […] Interstitial lung diseases […] Pulmonary edema […] Pneumonitis […] Metastases […]	The differential diagnoses do not consider the sparing of the periphery (as stated in the prompt).
[Radiography] The opacities are rather central and spare the periphery, the subpleural portions look normal. In addition, the opacifications are relatively small, but numerous and sometimes confluent. What are the three most likely differential diagnoses?	In a 55-year-old male patient with persistent cough and dyspnea, especially with physical exertion, and central, small-patchy, sometimes confluent infiltrates that spare the periphery and subpleural portions, the following differential diagnoses could be considered: Pneumoconiosis … Sarcoidosis … Bronchiolitis obliterans organizing pneumonia (BOOP) / cryptogenic organizing pneumonia (COP) […]	The differential diagnoses do not present with pulmonary opacifications that spare the periphery.
[CT] What cardiac or vascular causes can intermittent chest pain and dyspnea in a young male patient have? What do these look like in a CT scan?	[…] Aortic Dissection: While this is rare in young people, it can occur in individuals with certain genetic disorders or connective tissue diseases. It causes severe, sudden chest pain and can also cause dyspnea. In a chest CT, you would see a “false glade” or “intimal tumor” in the aorta. […]	Aortic dissection is usually described as a flap-like tear of the inner lining of the aorta with the creation of a false lumen. “False glade” or “intimal tumor” are incorrect descriptions.
[CT] An 18-year-old male patient presents with intermittent chest pain and dyspnea. The CT scan shows that the right coronary artery runs between the aorta and proximal pulmonary trunk. Can this explain the symptoms? What is this variant called?	The description of the right coronary artery running between the aorta and the pulmonary trunk suggests a condition called “Anomalous Origin of the Right Coronary Artery from the Left Sinus” (ARCAPA). However, the description of the coronary artery running between the aorta and the pulmonary trunk more closely matches another anomaly known as interarterial course or “coronary artery anomaly with interarterial course”. In this anomaly, the coronary artery normally originates from the aortic root, but runs between the aorta and the pulmonary artery. […]	First, the same diagnosis is provided twice. Second, “Anomalous Origin of the Right Coronary Artery from the Left Sinus” is not called “ARCAPA”, but “AAORCA”. “ARCAPA” refers to “Anomalous Right Coronary Artery Origin from Pulmonary Artery”.
[CT] Smoothly bordered liver lesion of about 10 cm with density values of 20-30 HU. Clinically fever and upper abdominal pain. Left hepatic cholestasis and a gallstone can be identified. What are the most likely diagnoses?	[…] A benign tumor such as a hemangioma or a liver adenoma would also be possible. The specific density values (HU) might suggest a lipoma or fatty degeneration.	Lipoma or fatty liver lesions are suggested based on specific density values. The prompt indicates 20-30 Hounsfield Units, while fat has approximately -120 to -90 Hounsfield Units.
[CT] What diseases should I consider with the following case description? “18-year-old male patient with severe abdominal pain, bloating, vomiting, and bloody stool”. In the CT scan, an ileus with a whirlpool sign can be seen. Give me the three most likely differential diagnoses as keywords without further explanation.	Volvulus, Mesenteric Ischemia, Intussusception	Intussusception does not present with the whirlpool sign but with the “target sign” and the “pseudokidney sign”.
[CT] A 32-year-old female patient presents with repeated pulmonary infections since childhood. The CT scan shows an almost non-aerated lung segment that is supplied by an artery from the celiac trunk. What are the three most likely differential diagnoses?	Differential diagnoses include […] Bronchiectasis […] Chronic Obstructive Pulmonary Disease (COPD) […]	Bronchiectasis and COPD are provided as differential diagnoses. However, they do not present with an unventilated lung segment supplied by an artery of the celiac trunk (as stated in the prompt).
[CT] What diseases should I consider with the following case description? “32-year-old female patient with repeated pulmonary infections since childhood”. In the CT scan, a partial vascular supply of the lung from the aorta can be seen. Give me the three most likely differential diagnoses as keywords without further explanation.	Ciliary Dyskinesia, Cystic Fibrosis, Bronchiectasis	The differential diagnoses do not consider the direct vascular supply from the aorta (as stated in the prompt).
[MRI] Signal characteristics of focal nodular hyperplasia (FNH), hepatic adenoma, and hemangioma.	[…] Liver Adenoma: In an MRI, a liver adenoma often appears less intense in T1-weighted sequences, while it looks more intense in T2-weighted sequences. After contrast agent injection, adenomas take up the contrast agent quickly in the early phase and then wash out in the later phases, causing them to appear less intense in later phases. […]	Hepatic adenoma is described as hypointense (T1-weighted images) and as displaying washout in the venous phase. Usually, hepatic adenomas are mildly hypointense or hyperintense (T1-weighted images) and do not display venous washout but rapid equilibration to near isointensity.
[MRI] 31-year-old female patient with recurring lower abdominal pain, especially after sexual intercourse and prolonged sitting. On MRI, the patient has dilated vessels in the pelvis. What are differential diagnoses?	[…] Potential differential diagnoses are […] 1) Pelvic Congestion Syndrome (PCS) […] 2) Endometriosis […] 3) Adenomyosis […] 4) Cysts or masses in the pelvic area […] 5) Interstitial Cystitis (Painful Bladder Syndrome) […] 6) Uterine prolapse […]	Endometriosis, adenomyosis, interstitial cystitis, and uterine prolapse are not primarily associated with dilated veins in the pelvis (as stated in the prompt).
[MRI] What hepatic space-occupying lesions are there, how often do they occur in relatively young women, and what do they look like on an MRI scan?	[…] Focal Nodular Hyperplasias (FNH): These are also benign lesions and can appear on MRI as well-defined, isointense or slightly hyperintense lesions on T1-weighted images and isointense or slightly hypointense lesions on T2-weighted images. […]	FNH is described as isointense or slightly hypointense on T2-weighted images, while -in fact- it is isointense or slightly hyperintense [18].
[MRI] A 41-year-old female patient presents with recurrent bilateral knee joint effusions. On MRI, a large knee joint effusion is visible with multiple oval dark portions within the effusion. Also, a septated cystic lesion is seen at the back of the knee. What are the three most likely differential diagnoses?	[…] Baker’s Cyst: This is a fluid-filled cyst that forms behind the knee joint and is typically associated with knee joint effusions. It can be visible on MRI and may appear as a dark area within the effusion.	Baker’s cysts are usually hyperintense in T2-weighted images and hypointense in T1-weighted images and located in the popliteal fossa while communicating with the joint. “Dark area within the effusion” is incorrect.
[MRI] Could the dark oval lesions within the joint effusion on T2-weighted images also be crystals as in gout?	[…] Additionally, the uric acid crystals typical of gout are usually not directly visible on a conventional MRI, they tend to lead to non-specific changes. Special MRI techniques such as dual-energy CT, however, can better visualize these crystals. […]	Dual-Energy CT was described as a “special MRI technique”.
[Angiography] What does a liver hemangioma look like in the angiogram, and how can I differentiate it from focal nodular hyperplasia or other liver cell tumors like hepatocellular carcinoma?	[…] In contrast, focal nodular hyperplasia (FNH) demonstrates a homogeneous contrast enhancement in the arterial phase with rapid washout of the contrast in the portal venous and delayed phase, leading to an isointense appearance compared to the surrounding normal tissue. A stellate scar tissue is often visible in the central region of the lesion. […]	FNH is described as a lesion with rapid washout in the venous phase, which is usually an imaging feature of HCC.
[Angiography] What can cause fever and elevated infection parameters after liver surgery with an in-situ PTCD (percutaneous transhepatic cholangiodrainage)? How do the possible causes present in an angiogram and compare to a drainage leak, a drainage misplacement, or a backlog of bile fluid?	[…] Abscess: An abscess in or near the liver could cause fever and elevated infection parameters. In an angiogram, an abscess would appear as a well-defined lesion with possible central necrosis or fluid collection.	Abscess may not be directly visualized in an angiogram. Peripheral hyperemia may be seen.
[Angiography] What arteries are in the thigh, and how do they run? How do they branch?	[…] The femoral artery runs along the front of the thigh and gives off several branches: Profunda femoris artery (deep artery of the thigh). […] After the profunda femoris artery branches off, the femoral artery continues its course towards the knee where it becomes the popliteal artery. This divides into the anterior tibial artery and posterior tibial artery, which supply blood to the lower leg and foot.	Common femoral and superficial femoral arteries are not differentiated. Fibular artery not mentioned.

Detailed are excerpts of the radiologists’ prompts and GPT -4’s responses, and the associated analyses. Prompts and responses have been redacted for conciseness, readability, and comprehension

### User experience

GPT-4’s utility was rated as fair (2.8 ± 0.4), satisfaction and trust as good (4.0 ± 0.6, 3.6 ± 0.8), and ease of use as excellent (4.5 ± 0.5).

## Discussion

Earlier studies investigating the potential value of GPT-4 in the clinic demonstrated excellent performance across various tasks and disciplines, particularly standardized medical examinations [6, 7, 16]. Recently, Kanjee et al used challenging clinicopathologic case conferences and found evidence of GPT-4’s ability to perform complex diagnostic reasoning [17]. These studies, however, relied on study settings not reflective of clinical reality, so little is known about GPT-4’s actual clinical value as an adjunct tool to the radiologist. Our study evaluated GPT-4’s utility for assisted image reading and interpretation and found modest improvements in diagnostic accuracy. Clinical experience was the preeminent factor determining diagnostic accuracy, while the modality and AI assistance had a more nuanced influence. AI assistance was beneficial by trend, yet its influence on diagnostic accuracy was statistically nonsignificant.

Our primary finding of improved diagnostic accuracy was valid for the ‘top-3 performance’. This finding is plausible given GPT-4’s broad, detailed knowledge of radiology. Notably, for radiography, we found a decline in diagnostic accuracy when radiographs were read with AI assistance. In modality-centered curricula (as in our hospital), radiography is the basic modality taught first. Our radiologists likely have broad radiographic knowledge, yet their performance was mixed. The adverse effects of AI interaction for radiography may be explained by overreliance and automation bias, which is the propensity to favor suggestions from automated systems. Sporadically, our radiologists cast wider nets of broader differentials under AI assistance instead of relying on their specific expertise. In contrast, greater improvements in CT, MRI, and angiography may be secondary to partially limited knowledge of these modalities, usually part of more advanced residency stages.

In several re-read imaging studies, the radiologists altered their interpretations. In 12 re-read studies, these conversions proved beneficial (i.e., incorrect to correct), while in 4 of 240 studies, the conversions were detrimental (i.e., correct to incorrect). For the former, differential diagnoses previously not mentioned were considered due to GPT-4’s response and involved primarily rare diagnoses with specific imaging features, such as pulmonary sequestration. For the latter, radiologists sporadically followed GPT-4’s guidance and agreed to the suggested and frequently generic differentials. This finding may be a potential sign of overreliance and automation bias. Regardless of their experience level, radiologists are prone to automation bias, and inexperienced radiologists are significantly more likely to follow the suggestions, even when blatantly false [18]. Undoubtedly, knowledgeable, skilled, and confident radiologists are key to mitigating these issues. AI may cover the width of potential diagnoses excellently, but it (still) requires trained radiologists to check consistency and reasoning.

Diagnostic accuracy dropped when defined more strictly as the ‘top-1 performance’. While the first diagnosis remained unchanged in most patients after interaction with GPT-4, we observed modality-specific effects on diagnostic accuracy that mirrored the ‘top-3 performance’ findings.

Our radiologists embraced AI assistance and used it in 78% of their readings. Prompt quantities indicate that our radiologists had conversational dialogues with GPT-4 when needed, as two or more prompts were used in a third of AI interactions. Prompts were primarily centered on possible differential diagnoses, which aligns well with a radiologist’s objective to keep rare differential diagnoses in mind when reading images. Prompting also focused on imaging features and information on pathology and anatomy. However, interactions with GPT-4 could not compensate for overlooked, ill-evaluated, and ill-described findings and provided valuable assistance only when specifically prompted. The complexity of describing specific findings can hardly be overcome for radiologists unfamiliar with a particular modality.

Diagnostic confidence was greater with AI assistance, yet this finding is only partially reflected by user experience ratings. While GPT-4’s ease of use was rated ‘excellent’, its utility was only ‘fair’, likely due to GPT-4’s inability to process images directly or provide an image search function for online repositories.

In line with previous reports [19], we found hallucinations that extended from disregarding diagnostically relevant information to providing false information. Undoubtedly, hallucinations are potentially harmful and even more so when considered against GPT-4’s purported trustworthiness, as our radiologists rated trust in GPT-4 as ‘good’. The clinical introduction of LLMs must be accompanied by appropriate safeguarding measures to ensure their accuracy and reliability and to prevent patient harm. Increasing user awareness, instituting quality checks by medical professionals, enhancing the LLM’s robustness against hallucinations, e.g., by process supervision instead of outcome supervision [20], and auditing adherence to regulatory standards may be parts of a strategy to counteract hallucinations effectively.

Our study has limitations. First, ten imaging studies were included per modality, which precludes clinical inference for particular modalities or pathologies and limits generalizability. Instead of assessing GPT-4’s value in every radiologic subdiscipline, we aimed to provide a preliminary and orientational evaluation across various preeminent imaging scenarios. Second, books or radiology-focused online references were prohibited per the study design to single out the effects of GPT-4. This restriction created a study setup unreflective of real-world radiologic practice. Future studies should compare established online resources, such as Radiopaedia and StatDx, with GPT-4 to determine their value in the reading room. Third, we did not institute a washout period between the reading sessions, which resulted in some studies being read on the same day and others more than 2 weeks apart. While performance metrics quantifying the impact of additional AI assistance on the benchmarked (unassisted) performance could be affected, we consider this approach acceptable nonetheless because (i) interpreting the studies unaided and then, if necessary, accessing assistance reflects the clinical practice and (ii) memory retention was likely high anyway given the select (and, in parts, memorable) patients and particular study conditions. Fourth, we refrained from assessing reporting times because our radiologists likely remembered the studies when re-reading them. Fifth, our evaluation used the May 2023 version of ChatGPT, which may only be partially reproduced by future versions, given the rapid evolution and undulating performance of different chatGPT versions [21]. Sixth, the GPT-4 utilization rates and patterns of general and subspecialty radiologists remain to be studied. Seventh, the LLM’s response is closely related to how it is prompted [22]. If and how the quality of GPT-4’s responses and the frequency of its hallucinations are affected by different prompting strategies must be studied in the future. Eighth, our sample size estimation must be, at best, considered a tentative approximation and should not be regarded as a precise measurement. Although the relatively small effect size of 0.2 was deliberate and informed by pertinent literature [14], it is important to acknowledge that the framework conditions in emerging research areas like AI-assisted diagnostics must be refined in the future. Ninth, streamlining the linear mixed-effect model improved the model’s interpretability, yet at the cost of reduced complexity. In prioritizing the reduction of variables to mitigate the risk of multicollinearity, we aimed to improve clarity and comprehensibility; yet, this approach may have oversimplified the complex interplay of factors affecting diagnostic accuracy and confidence.

## Conclusion

In conclusion, our study suggests that GPT-4 is a clinically useful adjunct tool that improves diagnostic accuracy slightly and diagnostic confidence significantly, and may partially mitigate the experience gap in radiologists. GPT-4 may facilitate more efficient and accurate diagnostic processes, yet it cannot replace a trained radiologist’s nuanced perception and critical thinking. Should GPT-4 or its successors be used in the clinical routine, safeguarding measures must be implemented to reduce hallucinations and their potential harm.

## Supplementary information

Electronic Supplementary Material

## Abbreviations

AI: Artificial intelligence
LLM: Large language model
PACS: Picture Archiving and Communication System
