# Supplementary material for: The virtual reference radiologist: comprehensive AI assistance for clinical image reading and interpretation
Source: Eur Radiol. 2024 Apr 16;34(10):6652–66. doi: 10.1007/s00330-024-10727-2 (PMC11399201; doi:10.1007/s00330-024-10727-2)
Supplement: Supplementary file 1 — Electronic Supplementary Material [file 330_2024_10727_MOESM1_ESM.pdf]

The Virtual Reference Radiologist: Comprehensive AI Assistance for Clinical Image Reading and Interpretation

ELECTRONIC SUPPLEMENTARY MATERIAL

Supplementary Text

Further Details on the Experimental Setup

The imaging studies were read on in-house radiology workstations with reporting monitors strictly adhering to clinical conditions. For each modality and session, questionnaires with dedicated reporting templates were designed using a dedicated and data privacy law-compliant online survey tool (s2survey.net [SoSci Survey GmbH, Munich, Germany], https://www.soscisurvey.de/en/index). First, the radiologists were broadly introduced to the study (**Supplementary Figure 1a**) through an example case. Second, the individual case descriptions (in the survey tool) and the respective studies (in the PACS) were presented individually and in a predetermined sequence. The radiologists were asked to provide up to three differential diagnoses (in the survey tool), ranked in descending order of probability, to explain the patient's symptoms, ideally specifying the severity of the condition (**Supplementary Figure 1b**). Third, the radiologists were asked to gauge the confidence level in their proposed diagnoses using a five-point Likert scale that ranged from 'very unsure' to 'very sure' for each patient (**Supplementary Figure 1c**). Diagnoses and confidence levels were logged automatically, and access to earlier pages was blocked after each patient.

The order of administration was (i) radiography, (ii) CT, (iii) MRI, and (iv) angiography. After completing the questionnaires unassistedly (session one), the radiologists re-read the same studies using AI assistance (session two). Time restrictions or a minimum washout period were not instituted, and the radiologists could re-read the imaging studies at their chosen time. However, they were instructed not to collect additional information or seek assistance on the patients, studies, or differential diagnoses between the readings. For the AI-assisted readings, GPT-4 was accessed online (https://chat.openai.com/) and operated as the chatGPT versions May 3rd, 12th, and 24th. A new chat session was started for each patient to avoid memory retention bias. The radiologists captured their complete interactions with GPT-4, and the investigators collected the (radiologists') prompts and (GPT-4's) responses.

Supplementary Figures

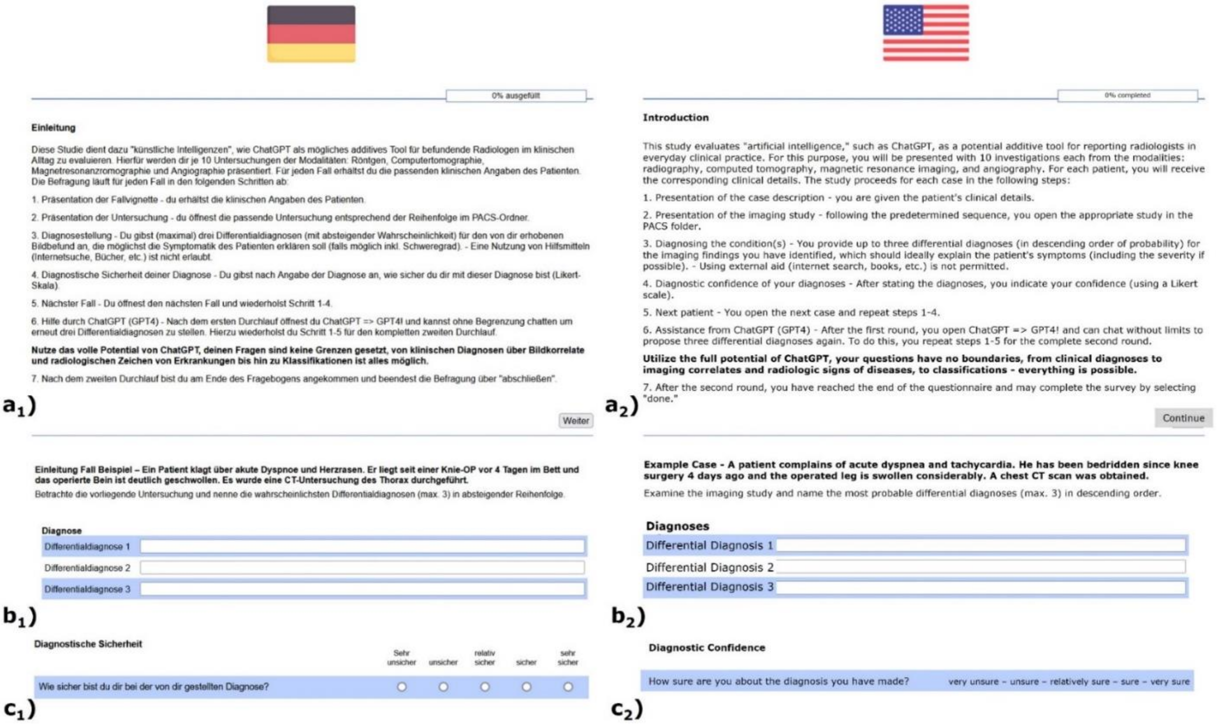

Supplementary Figure 1: Survey Tool and Questionnaire Details. Following the general introduction to the study (a_1 [English translation a_2]), the radiologists were asked to provide up to three differential diagnoses, ranked in descending order of probability and using the itemized reporting template (b_1 [English translation b_2]). Subsequently, the subjective confidence level for the particular imaging study was rated on a five-point Likert scale ranging from ‘very unsure’ to ‘very sure’ (c_1 [English translation c_2]).

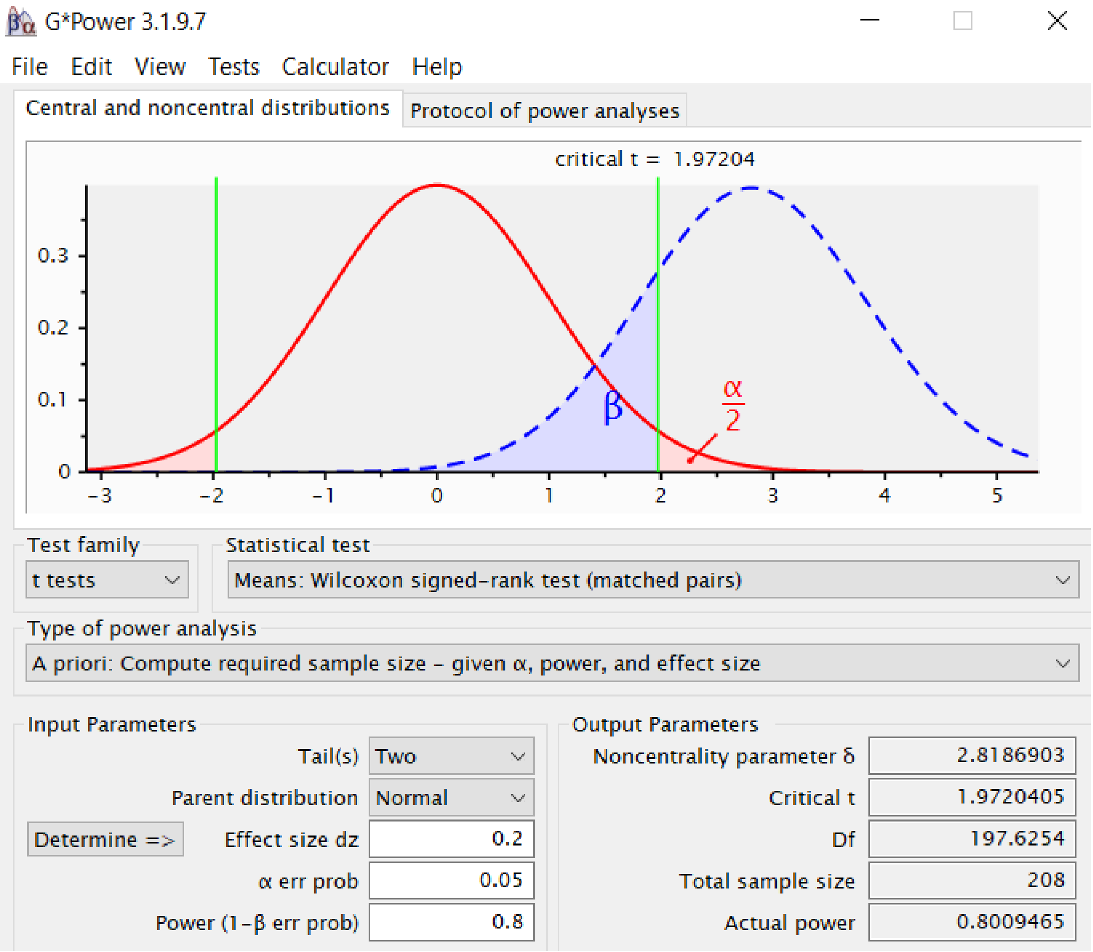

Supplementary Figure 2: Screenshot of the sample size calculation using G*Power software.

Supplementary Tables

Supplementary Table 1: Total Counts of Correct and Incorrect Diagnoses and Diagnostic Accuracy as a Function of Experience Level and Imaging Modality for AI-Assisted and Unassisted Readings When Considering the 'Top-3 Performance', i.e., the Count of Correct Diagnoses Among the Three Differential Diagnoses.
Presented are the aggregated counts of correct and incorrect diagnoses and diagnostic accuracies (calculated as the number of correct diagnoses divided by the number of correct and incorrect diagnoses) for radiologists with different levels of experience (summarized across all modalities), for each imaging modality (summarized across all experience levels), and for the different combinations of experience level and imaging modality. Counts and diagnostic accuracies are further stratified as AI-assisted or unassisted.

Experience Level	Imaging Modality	AI-Assisted Readings			Unassisted Readings		
		Correct [n]	Incorrect [n]	Diagnostic Accuracy [%]	Correct [n]	Incorrect [n]	Diagnostic Accuracy [%]
Advanced		71	9	89	70	10	88
Moderate		62	18	78	63	17	79
Low		55	25	69	48	32	60
	Angiography	48	12	80	46	14	77
	CT	54	6	90	51	9	85
	MRI	42	18	70	39	21	65
	Radiography	44	16	73	45	15	75
Advanced	Angiography	17	3	85	16	4	80
Advanced	CT	20	0	100	20	0	100
Advanced	MRI	17	3	85	17	3	85
Advanced	Radiography	17	3	85	17	3	85
Moderate	Angiography	17	3	85	17	3	85
Moderate	CT	18	2	90	18	2	90
Moderate	MRI	13	7	65	12	8	60
Moderate	Radiography	14	6	70	16	4	80
Low	Angiography	14	6	70	13	7	65
Low	CT	16	4	80	13	7	65
Low	MRI	12	8	60	10	10	50
Low	Radiography	13	7	65	12	8	60
**All**	**All**	**188**	**52**	**78**	**181**	**59**	**75**

Supplementary Table 2: Total Counts of Correct and Incorrect Diagnoses and Diagnostic Accuracy as a Function of Experience Level and Imaging Modality for AI-Assisted and Unassisted Readings When Considering the 'Top-1 Performance', i.e., the Count of Correct Diagnoses provided as the First Differential Diagnosis.
Presented are the aggregated counts of correct and incorrect diagnoses and diagnostic accuracies (calculated as the number of correct diagnoses divided by the number of correct and incorrect diagnoses) for radiologists with different levels of experience (summarized across all modalities), for each imaging modality (summarized across all experience levels), and for the different combinations of experience level and imaging modality. Counts and diagnostic accuracies are further stratified as AI-assisted or unassisted.

Experience Level	Imaging Modality	AI-Assisted Readings			Unassisted Readings		
		Correct [n]	Incorrect [n]	Diagnostic Accuracy [%]	Correct [n]	Incorrect [n]	Diagnostic Accuracy [%]
Advanced		63	17	79	63	17	79
Moderate		53	27	66	52	28	65
Low		47	33	59	39	41	49
	Angiography	40	20	67	40	20	67
	CT	49	11	82	46	14	77
	MRI	37	23	62	30	30	50
	Radiography	37	23	62	38	22	63
Advanced	Angiography	14	6	70	15	5	75
Advanced	CT	18	2	90	18	2	90
Advanced	MRI	17	3	85	15	5	75
Advanced	Radiography	14	6	70	15	5	75
Moderate	Angiography	14	6	70	14	6	70
Moderate	CT	16	4	80	16	4	80
Moderate	MRI	11	9	55	9	11	45
Moderate	Radiography	12	8	60	13	7	65
Low	Angiography	12	8	60	11	9	55
Low	CT	15	5	75	12	8	60
Low	MRI	9	11	45	6	14	30
Low	Radiography	11	9	55	10	10	50
**All**	**All**	**163**	**77**	**68**	**154**	**86**	**64**

Supplementary Table 3: Results of the Simplified Generalized Linear Mixed-Effects Model Analysis for the 'Top-3 Performance' and 'Top-1 Performance'.

The coefficients and p-values for each predictor are presented, quantifying its effect on diagnostic accuracy. The coefficients represent the change in the log odds of giving the correct diagnosis for each category compared to the reference categories, i.e., advanced (experience level), angiography, and AI-assisted. Coefficients are given as Mean [95 % Confidence Interval]. Significant associations are indicated in **bold type**. Normality testing (D'Agostino's K^2 test) indicated that the residuals conformed to an (approximate) normal distribution with a test statistic of 0.248 (p=0.883).

**Predictor**	**Top-3 Performance**		**Top-1 Performance**	
	**Coefficient**	***p*-value**	**Coefficient**	***p*-value**
**Experience_Level[Low]**	-1.46 [-2.05; -0.87]	**<0.001**	-1.20 [-1.7; -0.7]	**<0.001**
**Experience_Level[Moderate]**	-0.75 [-1.37; -0.14]	**0.017**	-0.69 [-1.19; -0.18]	**0.008**
**Modality[CT]**	0.69 [-0.02; 1.40]	0.056	0.67 [0.08; 1.27]	**0.027**
**Modality[MRI]**	-0.59 [-1.19; 0.01]	0.052	-0.49 [-1.02; 0.05]	0.077
**Modality[Radiography]**	-0.25 [-0.86; 0.37]	0.435	-0.19 [-0.74; 0.35]	0.488
**Assistance_Status[Unassisted]**	-0.18 [-0.62; 0.27]	0.428	-0.18 [-0.58; 0.21]	0.366

Read the table as follows: For the 'top-3 performance', "Experience_Level[Low]" has a mean coefficient of -1.46, which indicates that the chance of providing the correct diagnosis is lower for a low-experience radiologist compared to an advanced-experience radiologist. $p < 0.001$ indicates the association to be significant. In contrast, "Modality[CT]" has a mean coefficient of +0.69, which indicates an increased, yet statistically not significant ($p = 0.056$) likelihood of a correct diagnosis when interpreting CT versus angiographic studies.

Supplementary Table 4: Effects of Experience Levels, Imaging Modality, and AI Assistance on Diagnostic Confidence

Given are the results of a linear mixed-effect model with coefficients and their associated 95% confidence intervals (mean [95 % Confidence Interval]), and p-values. Predictors with significant effects on diagnostic confidence scores are highlighted in **bold type**. Advanced experience level, angiography, and AI-assisted (readings) served as the reference categories. Normality testing (D'Agostino's K^2 test) indicated that the residuals conformed to an (approximate) normal distribution with a test statistic of 5.4812 (p=0.0645).

Predictor	Coefficient	*p*-value
**Experience_Level[Low]**	0.40 [-0.65; 1.45]	0.456
**Experience_Level[Moderate]**	1.71 [0.85; 2.58]	**<0.001**
**Modality[CT]**	0.17 [-0.44; 0.77]	0.590
**Modality[MRI]**	-0.88 [-1.44; -0.31]	**0.003**
**Modality[Radiography]**	-0.06 [-0.46; 0.35]	0.777
**Assistance_Status[Unassisted]**	-0.49 [-0.66; -0.33]	**<0.001**

Supplementary Table 5: 'Misinterpretations' by GPT-4. Detailed are excerpts of the radiologists' prompts and GPT -4's responses, and the associated analyses. Prompts and responses have been redacted for conciseness, readability, and comprehension.

Radiologist's Prompt	GPT -4's Hallucinatory Response	Analysis of Hallucination
[Angiography] Dysfunction of the port catheter with missing contrast medium near the tip. What is the diagnosis?	It sounds like there may be an issue with the port catheter, as the contrast medium is leaking near the tip of the catheter. […]	"Missing contrast medium" is misunderstood and interpreted as "leakage".
[Angiography] How do I distinguish HCC from other liver tumors in angiography? Please elaborate on distinguishing criteria.	Hepatocellular carcinoma (HCC) is one of the most common types of liver tumors and can exhibit characteristic features in various medical imaging studies, including angiography. […] Hypervascularity in the arterial phase […] Washout in the portal-venous or late phase […] Arterioportal shunts […] Necrosis or hemorrhage […]	Only imaging criteria of HCC are mentioned, while distinguishing criteria (versus other hepatic lesions) were explicitly asked.
